# Efficacy and safety of vedolizumab and infliximab treatment for immune-mediated diarrhea and colitis in patients with cancer: a two-center observational study

**DOI:** 10.1136/jitc-2021-003277

**Published:** 2021-11-17

**Authors:** Fangwen Zou, David Faleck, Anusha Thomas, Jessica Harris, Deepika Satish, Xuemei Wang, Aline Charabaty, Marc S Ernstoff, Isabella C Glitza Oliva, Stephen Hanauer, Jennifer McQuade, Michel Obeid, Amishi Shah, David M Richards, Elad Sharon, Jedd Wolchok, John Thompson, Yinghong Wang

**Affiliations:** 1 Department of Oncology, Second Xiangya Hospital, Changsha, Hunan, China; 2 Department of Gastroenterology, Hepatology & Nutrition, The University of Texas MD Anderson Cancer Center, Houston, Texas, USA; 3 Department of Gastroenterology, Hepatology and Nutrition, Memorial Sloan Kettering Cancer Center, New York, New York, USA; 4 Department of Biostatistics, The University of Texas MD Anderson Cancer Center, Houston, Texas, USA; 5 Department of Gastroenterology, Johns Hopkins University, Washington, DC, USA; 6 Division of Cancer Treatment and Diagnosis, National Cancer Institute, Bethesda, Maryland, USA; 7 Department of Melanoma Medical Oncology, The University of Texas MD Anderson Cancer Center, Houston, Texas, USA; 8 Department of Medicine, Northwestern University, Chicago, Illinois, USA; 9 Department of Medicine, Centre Hospitalier Universitaire Vaudois, Lausanne, Switzerland; 10 Department of Genitourinary Medical Oncology, The University of Texas MD Anderson Cancer Center, Houston, Texas, USA; 11 Human Oncology and Pathogenesis Program, Department of Medicine, Memorial Sloan Kettering Cancer Center, New York, New York, USA; 12 University of Washington, Seattle Cancer Care Alliance, Fred Hutchinson Cancer Research Center, Seattle, Washington, USA

**Keywords:** cytotoxicity, immunologic, immunotherapy, inflammation, melanoma

## Abstract

**Background:**

Current treatment guidelines for immune-mediated diarrhea and colitis (IMDC) recommend steroids as first-line therapy, followed by selective immunosuppressive therapy (SIT) (infliximab or vedolizumab) for refractory cases. We aimed to compare the efficacy of these two SITs and their impact on cancer outcomes.

**Methods:**

We performed a two-center, retrospective observational cohort study of patients with IMDC who received SITs following steroids from 2016 to 2020. Patients’ demographic, clinical, and overall survival data were collected and analyzed.

**Results:**

A total of 184 patients (62 vedolizumab, 94 infliximab, 28 combined sequentially) were included. The efficacy of achieving clinical remission of IMDC was similar (89% vs 88%, p=0.79) between the two groups. Compared with the infliximab group, the vedolizumab group had a shorter steroid exposure (35 vs 50 days, p<0.001), fewer hospitalizations (16% vs 28%, p=0.005), and a shorter hospital stay (median 10.5 vs 13.5 days, p=0.043), but a longer time to clinical response (17.5 vs 13 days, p=0.012). Longer durations of immune checkpoint inhibitors treatment (OR 1.01, p=0.004) and steroid use (OR 1.02, p=0.043), and infliximab use alone (OR 2.51, p=0.039) were associated with higher IMDC recurrence. Furthermore, ≥3 doses of SIT (p=0.011), and fewer steroid tapering attempts (p=0.012) were associated with favorable overall survival.

**Conclusions:**

Treatment with vedolizumab as compared with infliximab for IMDC led to comparable IMDC response rates, shorter duration of steroid use, fewer hospitalizations, and lower IMDC recurrence, though with slightly longer time to IMDC response. Higher number of SIT doses was associated with better survival outcome, while more steroid exposure resulted in worse patient outcomes.

## Introduction

The advent of immune checkpoint inhibitors (ICIs) has drastically transformed the management of advanced cancers.^1^ However, immune checkpoint blockade can induce broad autoimmunity beyond selective upregulation of tumor-antigen specific T-cells, which can impact other organ systems leading to a variety of immune-related adverse events (irAEs). Immune-mediated diarrhea and colitis (IMDC) is one of the most common irAEs and is often the main reason for ICI discontinuation and morbidities.^2^

Current guidelines for IMDC recommend steroids as first-line treatment, followed by selective immunosuppressive therapy (SIT) for steroid-refractory cases.^3^ However, protracted courses of high-dose steroids can lead to numerous unfavorable complications, and may negatively influence cancer response to ICI therapy and overall cancer prognosis.^4^ Therefore, early introduction of SIT in the course of IMDC to reduce steroid usage and hasten symptom resolution has been proposed as a preferred management strategy.^5^

The two commonly used SITs for IMDC are infliximab (IFX) and vedolizumab (VDZ). IFX is a chimeric antitumor necrosis factor-alpha (TNFα) monoclonal antibody^6^ that acts by immunosuppression, broadly dampening TNFα’s role in the immune response. In contrast, VDZ is a gut-selective humanized anti-α_4_β_7_ monoclonal antibody that binds to the gut leukocyte adhesion molecule α_4_β_7_-integrin and prevents lymphocyte infiltration in the gut.^7^ Both SITs are approved for treating inflammatory bowel disease (IBD),^8^ and both have shown favorable clinical outcomes in recent small studies of IMDC.^9 10^

Despite the existing data on SITs in treating IMDC, there has not been a direct comparison of the efficacy and safety of these two agents. In addition, the impact of steroids and different SITs on cancer treatment response remains unclear. Therefore, the selection of IFX and/or VDZ is often based on the clinician’s discretion, drug availability, and financial limitation. Given the difference in the underlying mechanisms of action, we hypothesized that VDZ would result in similar efficacy of IMDC treatment, less AEs, and superior cancer outcome and survival.

To address this hypothesis, we conducted a two-center retrospective comparative study of patients with IMDC who received either IFX or/and VDZ after first-line corticosteroid therapy. This study will help determine and compare the clinical efficacy and safety of IFX and VDZ in patients with cancer with IMDC and provide additional reference for clinicians managing IMDC.

## Methods

### Patient cohort

Patients’ informed consent was waived given retrospective design. Included patients were ≥18 years of age that received any ICI and developed IMDC that was treated with steroids and IFX and/or VDZ between March 2016 and March 2020. Patients who had IBD, mesenteric ischemia before ICI treatment, or ICI resumption after IMDC during the study period were excluded. The details of patient distribution are shown in online supplemental table 1. Data collected at Memorial Sloan Kettering Cancer Center were deidentified and centralized at MD Anderson Cancer Center (MDACC) for final analysis.

10.1136/jitc-2021-003277.supp1 Supplementary data

### Clinical data collection

All patient data were obtained from institutional electronic medical charts and pharmacy databases. Demographic data, cancer and IMDC-related information were extracted. IMDC diagnosis was made by the treating physician based on the exclusion of other etiologies. Complications of immunosuppressants were reported between steroid initiation to 30 days after last dose of any immunosuppressant (steroid or SITs). For patients who received both agents, SITs were given sequentially but not concurrently during the same IMDC event or its recurrence, and the switch was due to treatment failure or AEs. The sequence of administration was at the discretion of treating physicians. Based on the SIT treatments received, patients were divided into three groups for analysis, IFX alone, VDZ alone and both combined sequentially. CTCAE v5.0^11^ was used to measure the severity of diarrhea and colitis symptoms. Endoscopic and histological evaluations were also recorded and categorized based on the criteria illustrated previously.^12^

### IMDC clinical outcomes assessment

Clinical outcomes included IMDC-related requirement for multiple hospitalizations or admission to an intensive care unit, duration of hospitalization, clinical remission or response of IMDC, IMDC recurrence, and cancer outcome and OS. IMDC clinical remission was defined as sustained resolution of diarrhea or colitis to grade 1 or lower. IMDC clinical response was defined as a sustained improvement of IMDC symptoms, but not achieving remission. IMDC recurrence was defined as recurrent symptoms beyond 1 month after the initial IMDC episode had resolved. The follow-up duration is administratively censored to maximal 45 months to alleviate the effect of follow-up duration variation among different cohorts. Cancer status was assessed according to the Immune-modified Response Evaluation Criteria in Solid Tumors or oncology note^13^ at IMDC onset and last follow-up.

### Statistical analysis

Statistical analyses were performed by SPSS V.24.0 software (SPSS). Continuous variables were presented as the mean and SD or median and IQR and compared using the Wilcoxon rank-sum test or Kruskal-Wallis test. Categorical variables were described by frequencies and percentages and compared by Fisher’s exact and χ^2^ tests. The Kaplan-Meier method and log-rank test were used to evaluate OS, defined as the duration from IMDC onset to last follow-up or death due to any cause. Univariate and multivariate Cox regression models were fit to assess the association between OS and patient characteristics. Risk factors for IMDC recurrence and cancer progression were assessed by logistic regression models with ORs and 95% CIs. All statistical tests were two sided, and p<0.05 was considered statistically significant.

## Results

### Patient baseline characteristics

A total of 184 patients (62 VDZ, 94 IFX, 28 combined sequentially) were included (online supplemental table 2). Our cohorts had a median age of 64 years, 118 patients (64%) were male, and 169 (92%) were Caucasian. Melanoma (34%) and genitourinary cancer (33%) were the most common malignancies. A total of 153 patients had endoscopic evaluation with confirmed histological inflammation. Fifty-one per cent of patients were treated with IFX alone, 34% with VDZ alone, and 15% were treated with combined SIT agents sequentially. The non-gastrointestinal (GI) organ irAEs are summarized in online supplemental table 2. The median follow-up duration was 14 months (IQR 8–27).

10.1136/jitc-2021-003277.supp2 Supplementary data

### Comparison of baseline and IMDC characteristic between IFX and VDZ-treated patients

With respect to cancer type, the IFX group had a higher proportion of melanoma (47% vs 16%, p<0.001) and less frequent use of programmed cell death 1 protein/ligand one inhibitors (43% vs 61%, p=0.041) (table 1). In contrast, the VDZ group had a shorter duration of steroid use (p<0.001), fewer steroid tapering attempts (p=0.016), fewer patients with multiple hospitalizations (p=0.005), and shorter length of cumulative hospital stay (p=0.043) (table 2). The endoscopic and histologic features are comparable between the two treatment groups. The rate of clinical remission from initial IMDC was equivalent, around 88%, although VDZ group was observed to have a delayed time to clinical response (18 vs 13 days, p=0.012), and a trend towards longer symptom duration (56 vs 50 days, p=0.054) during the initial IMDC episode. Further, we found that, the VDZ alone group had the lowest rate of IMDC recurrence compared with the IFX alone group and patients received combined SITs sequentially (14% vs 29% vs 25%, p=0.008, respectively; online supplemental table 3).

**Table 1:** Baseline clinical characteristics of patients on treatment of vedolizumab and infliximab alone (N=156)

Characteristic	Vedolizumab n=62	Infliximab n=94	P value
Median age at IMDC, years (IQR), n=156	63 (49–71)	65 (50–73)	0.634
Male sex, no (%)	41 (66)	58 (62)	0.358
White race, no (%)	58 (94)	86 (91)	0.736
Median Charlson Comorbidity Index at colitis diagnosis, points (IQR), n=156	8 (7–9)	8 (6–9)	0.931
Cancer type, no (%)			<0.001
Melanoma	10 (16)	44 (47)	
Genitourinary cancer	23 (37)	26 (28)	
Lung cancer	10 (16)	16 (17)	
Others	19 (31)	8 (8)	
Cancer stage, no (%)			0.245
Stage III	12 (19)	14 (15)	
Stage IV	50 (81)	80 (85)	
Checkpoint inhibitor type, no. (%)			0.041
Anti-CTLA-4	6 (10)	17 (18)	
Anti-PD-(L)1	38 (61)	40 (43)	
Combination	18 (29)	37 (39)	
Median follow-up duration, months (IQR), n=156	15 (11–26)	14 (7–30)	0.542

CTLA-4, cytotoxic T-lymphocyte-associated protein 4; IMDC, immune-mediated diarrhea and colitis; PD-(L)-1, programmed cell death 1 protein/ligand.

**Table 2:** IMDC-related characteristics in patients treated with vedolizumab and infliximab alone (N=156)

Characteristic	Vedolizumab n=62	Infliximab n=94	P value
Median length of ICI treatment, days (IQR), n=156	73 (28–247)	70 (28–151)	0.846
Median time from ICI initiation-IMDC onset, days (IQR), n=156	104 (53–212)	96 (33–188)	0.065
No of ICI treatments before IMDC, mean (SD)	7 (8)	7 (6)	0.235
Diarrhea grade, no (%)			0.583
1–2	20 (32)	33 (35)	
3–4	42 (68)	61 (65)	
Colitis grade, no (%)			0.246
1–2	30 (48)	57 (61)	
3–4	32 (52)	37 (39)	
Median duration of initial IMDC symptoms, days (IQR), n=156	56 (27–80)	50 (31–69)	0.054
Endoscopy evaluation, no (%)	60 (96)	65 (69)	<0.001
Endoscopic presentation, no (%)			0.265
Mucosal ulceration	17 (28)	19 (29)	
Non-ulcerative inflammation	32 (54)	37 (57)	
Normal	11 (18)	9 (14)	
Histology features–no (%)			0.105
Acute active colitis	12 (20)	24 (37)	
Chronic active colitis	35 (58)	31 (48)	
Microscopic colitis	13 (22)	10 (15)	
IV steroids, no (%)	36 (58)	58 (62)	0.237
Median duration of steroids for initial IMDC, days (IQR), n=156	35 (27–43)	51 (41–68)	<0.001
No of steroid tapering attempts prior to SIT use, median (IQR), n=156	1 (1–3)	2 (2–3)	0.016
Median duration from IMDC to first dose of SIT- days (IQR), n=156	11 (9–48)	23 (19–37)	<0.001
No of SIT doses (%)			<0.001
1–2	21 (34)	77 (82)	
≥3	41 (66)	17 (18)	
Doses of SIT, mean (SD)	3 (2)	2 (1)	<0.001
Median duration from first dose of SIT to symptom remission or improvement to grade 1, days (IQR), n=138	18 (10–40)	13 (8–29)	0.012
Hospitalization, no (%)	40 (65)	67 (71)	0.367
Median duration of hospitalization, days (IQR), n=107	10 (5–15)	14 (8–19.8)	0.043
Multiple hospitalizations, no (%)	10 (16)	26 (28)	0.005
Clinical remission, no (%)	55 (89)	83 (88)	0.785
Recurrent IMDC, no. (%)	8 (14)	27 (29)	0.007
Immunosuppressant-associated infection, no (%)	12 (19)	23 (25)	0.184

ICI, immune checkpoint inhibitor; IMDC, immune-mediated diarrhea and colitis; IV, intravenous; SIT, selective immunosuppressive therapy.

### Risk factors for IMDC recurrence

We observed the following risk factors for higher IMDC recurrence (table 3): longer duration of ICI (OR 1.01, p=0.004), later onset of IMDC (OR 1.01, p=0.002), longer duration of steroids (OR 1.02, p=0.043), and IFX monotherapy (OR 2.51, p=0.039). Multivariate logistic regression analysis confirmed that later onset of IMDC after ICI initiation (OR 1.09, p=0.002) and longer duration of steroid use (OR 1.48, p=0.033) were both risk factors for higher recurrence.

**Table 3:** Univariate and multivariate logistic regression analysis for risk factors for IMDC recurrence

Variable	Univariate analysis			Multivariate analysis		
	OR	95% CI	P value	OR	95% CI	P value
Charlson Comorbidity Index	1.03	0.87 to 1.21	0.775			
Duration of ICI treatment	1.01	1.00 to 1.05	0.004			
ICI type						
Anti-CTLA-4		Reference				
Anti-PD-(L)−1	1.6	0.43 to 3.09	0.771			
Combination	0.49	0.16 to 1.49	0.210			
Duration from ICI to IMDC onset	1.09	1.03 to 1.15	0.002	1.09	1.03 to 1.15	0.002
Duration of colitis symptoms	1.01	0.99 to 1.02	0.487			
Diarrhea grade 3–4 vs 1–2	1.06	0.51 to 2.21	0.879			
Colitis grade 3–4 vs 1–2	0.98	0.48 to 1.94	0.911			
Overall duration of steroids	1.44	1.01 to 2.06	0.043	1.48	1.03 to 2.12	0.033
IV steroids	1.59	0.76 to 3.33	0.223			
Type of SIT						
Vedolizumab		Reference				
Infliximab	2.51	1.05 to 5.99	0.039			
Both sequentially	2.86	0.89 to 9.19	0.078			
Dose of SIT	1.04	0.91 to 1.18	0.613			
Time from IMDC to first dose of SIT	1.00	0.98 to 1.01	0.064			

CTLA-4, cytotoxic T-lymphocyte-associated protein 4; ICI, immune checkpoint inhibitor; IMDC, immune-mediated diarrhea and colitis; IV, intravenous; PD-(L)−1, programmed cell death 1 protein/ligand; SIT, selective immunosuppressive therapy.

### Cancer progression among different treatment regimens and its risk factors

At the time of IMDC diagnosis, the rate of cancer progression was comparable among all three groups (24% vs 36% vs 18%, p=0.107). At the time of last follow-up, the rate of cancer progression had increased in all groups, but patients in IFX group had a higher rate compared with the other two groups (54% vs 34% vs 43%, respectively, p=0.042, online supplemental figure 1). These findings were further confirmed by a subgroup analysis among 163 patients who had a comparable follow-up time window (2017–2020) (online supplemental table 4).

10.1136/jitc-2021-003277.supp3 Supplementary data

Univariate analysis showed that higher Charlson Comorbidity Index (CCI) (OR 1.20, p=0.012), longer duration of steroids (OR 1.01, p=0.007), and IFX monotherapy (OR 2.32, p=0.013) were associated with a higher risk of cancer progression. Multivariate logistic regression analysis confirmed the associations between cancer progression and higher CCI (OR 1.21, p=0.020) and IFX monotherapy (OR 5.24, p<0.001). Melanoma-specific cancer type was associated with a lower probability of cancer progression (OR 0.18, p=0.005; online supplemental table 5).

### Overall survival analyses

Patients who received VDZ monotherapy had more favorable OS than those received IFX monotherapy (p=0.027, online supplemental figure 2A). Patients who received combined SITs sequentially had an intermediate OS. Fewer steroid tapering attempts were associated with better OS (online supplemental figure 2B, p=0.012). Similar outcome was also observed in patients who received ≥3 SIT doses (p=0.011, online supplemental figure 2C). These findings were further confirmed by a subgroup analysis among the 163 patients with a comparable follow-up window (2017–2020) (online supplemental figure 3A). Multivariate Cox regression analysis demonstrated that higher CCI (HR 1.18, p=0.009) and IFX monotherapy (HR 2.04, p=0.014) were associated with worse OS (online supplemental table 6).

10.1136/jitc-2021-003277.supp4 Supplementary data

In terms of association with different cancer types, superior OS from VDZ among patients with genitourinary and lung cancer was observed when compared with the IFX group (p=0.041 and p=0.046, respectively, online supplemental figure 4A, B). The link between VDZ and longer OS was further confirmed with a subgroup analysis among patients with a similar follow-up window (2017–2020) (online supplemental figure 3B–D).

10.1136/jitc-2021-003277.supp5 Supplementary data

### AEs related to immunosuppressant treatment

Infection was one of the most frequent AEs associated with immunosuppressant (steroid and SIT) therapy, accounting for 24% of all AEs. The infections reported in our study included GI (41%) and urinary tract infections (37%), pneumonia (7%), periodontitis (7%), bacteremia (5%), and others. Overall, the rates of infection were similar between patients who had long and short durations of steroid exposure or received fewer and more steroid treatment courses (online supplemental figure 5A, B). However, the substantial rise of infection was evident in the IFX group with more steroid treatment courses (19% vs 39%, p=0.045).

## Discussion

This study is the first large, two-center comparative study of the clinical efficacy, AEs and cancer outcomes between IFX and VDZ treatments for patients with IMDC. We observed that patients treated with VDZ as compared with IFX had comparable IMDC response rates, shorter duration of steroid use, fewer hospitalizations, and lower IMDC recurrence, though with slightly longer time to IMDC response.

While IMDC is the second most frequent irAEs, robust evidence for various treatment regimens are lacking, particularly in reference to steroid sparing therapies such as IFX and VDZ.^2^ IFX is more commonly used for IMDC, likely due to several factors. First, as the initial FDA approved SIT in 1998 for IBD, it was also the effective biological agent used early to manage steroid-refractory IMDC. VDZ was not approved for clinical use in IBD until 2014 and therefore is less studied. Furthermore, the advent of biosimilars and large observational cohort studies in IBD showing that an IFX biosimilars has comparable efficacy and safety with the originator and more appealing financial cost could potentially affect the management of IMDC as well.^14 15^ Finally, most oncology guidelines recommended VDZ only for patients with contraindications or non-response to IFX,^16^ and so were often not prescribed as first line SIT nor readily covered by insurance. This maybe in part due to the fact that these guidelines are written primarily by oncologists with little experience with anti-integrin therapy.

IFX has been studied extensively in the IBD population and can effectively induce and maintain enteric mucosal healing.^17^ However, given its systemic mode of action, this drug is also associated with serious AEs such as infections and malignancy.^18^ It is important to recognize that the vast majority of studies of IFX provide data on the safety of the long-term TNF-ɑ blockade in a non-cancer population. In the field of IMDC, the limited evidence showing IFX’s association with decreased survival comes from the Dutch Melanoma Treatment Registry.^19^ Unlike the broad mechanism of IFX, VDZ can selectively inhibit the interaction between leukocytes and the intestinal vasculature and prevent the influx of inflammatory cells, which mediate the inflammatory process in IMDC.^20^ Given its highly gut-selective mode of action, long-term VDZ treatment has a favorable safety profile with low incidence of serious infections and malignancies.^21^ An integrated study on the safety of VDZ based on six IBD trials that involved 2830 patients summarized no increased risk of infections as compared with placebo as well as low rates of malignancies (<1%).^22^

Though there are multiple case series of IFX and VDZ in treating IMDC,^10 23^ our retrospective comparative analysis of these two agents in a cancer population with IMDC is the first of its kind. While both these agents are used routinely in the management of IMDC, it is important to note that most of the available data are extrapolated from clinical trials in patients with IBD. Despite the similarities between IMDC and IBD, there are substantial differences that may impact their relative efficacy and safety, for example, the presence of underlying cancer, and the shorter duration of SIT therapy for IMDC, and underscore the importance of comparative data focused on IMDC.

The majority of our sample was comprised of patients with advanced cancer and severe IMDC. Of note, there were no significant differences in IMDC severity between the SIT treatment groups as assessed by CTCAE grade and endoscopic/histologic findings. Nonetheless, patients treated with IFX received a significantly longer duration of steroid treatment, more steroid treatment courses, and had higher rates of IMDC recurrence as compared with the VDZ treatment group. Together, these observations appear to favor the use VDZ over IFX for treatment of steroid-refractory IMDC, though at the expense of a slightly longer latency to clinical response. The slower response time to VDZ mirrors observations in IBD, though robust data regarding response time are limited.^24 25^

However, there were notable differences in prescribing patterns between IFX and VDZ that may have influenced efficacy and clinical outcomes as well. We observed a delay in initiation of IFX as compared with VDZ in the colitis disease course (median of 23 vs 11 days). Prior evidence suggests that early SIT introduction can result in a higher efficacy against IMDC, which may favor the VDZ-treated group.^5^ Moreover, the IFX group received fewer doses of SIT than did the VDZ group, which may have contributed to the significantly higher IMDC recurrence seen with IFX. This observation supports our previous study on the contributing factors associated with lower IMDC recurrence, with higher number of SIT doses correlating with lower recurrence rates.^5^ Some of the outcome differences, therefore, may reflect the wide variances in practice pattern among different providers in terms of SIT choice, timing of SIT initiation as well as doses of SIT administered, rather than differences in the drug mechanism of action alone.

On scrutinizing our subgroup that received combined SITs sequentially, 25 patients (89%) switched from IFX to VDZ, while 11% switched from VDZ to IFX, and 68% of patients in this group achieved IMDC remission. For this challenging group of patients refractory to one or two lines of SIT, alternative therapies such as FMT^26^ or other novel agents can be considered. Ustekinumab^27^ and tofacitinib^28^ are employed as induction and maintenance therapy for IBD by inhibition of interleukin 12/23 and Janus Kinase, respectively. These agents require further evaluation in the management of IMDC, and current evidence supporting their use is limited to case series and reports.^29–31^

We observed an overall infection rate of 24% in our study. These results are in keeping with those from a prior study that noted a significantly lower rate of infections (30%) in patients treated with a combination of SIT and steroid compared with long duration of steroid alone (51%).^32^ We suspect that SIT therapy overcame steroid dependence and duration to an extent and potentially negated any significant difference in AEs that might have resulted from prolonged systemic steroids.

We also assessed cancer outcomes among the patients in this study as an exploratory analysis, with the understanding that retrospective, uncontrolled data from a heterogenous group of patients with IMDC poses significant limitations. We observed a significantly higher rate of cancer progression at last follow-up in the IFX-treated group and inferior long-term OS, despite similar baseline cancer status at IMDC onset. While we speculated that considerable exposure to steroids in the IFX group may have contributed to the worse cancer outcomes, this hypothesis was not supported by the outcome of the group who received combined SITs sequentially, which notably received the most intravenous steroids and had equivalent steroid treatment courses. Nonetheless, overall duration of steroid exposure was associated with worse survival in univariate analysis, an observation that has been made in studies of other irAEs as well.^33 34^ Interestingly, when accounting for cumulative doses of SIT treatments, it appeared that more doses were beneficial to patients’ overall survival. This observation may reflect the extended colitis disease course in these patients and signs of prolonged ICI effect. This finding is consistent with a previous study showing an association between chronic IMDC and favorable cancer outcome.^35^

Undoubtedly, other confounding factors for example, different tumor biology and burden, differences in ICI regimens, selection of different SIT agent and timing of endoscopy evaluation should also be taken into consideration when interpreting the cancer outcomes and survival data, which we view as hypothesis-generating rather than conclusive data. We are hopeful that the upcoming prospective randomized clinical trial (NCT04407247) between IFX and VDZ to be conducted at MDACC will further delineate the role of each SIT in cancer prognosis. Taking all the pros and cons into consideration, we speculate that a combination strategy of using one dose of IFX for quicker induction followed by long-term VDZ maintenance could potentially minimize the unfavorable effect of IFX and maximize the beneficial aspect of VDZ for both IMDC and cancer outcomes.

Our study has several limitations. First, this is a retrospective study with all the inherent limitations that stem from this study design. Second, because the study is from two tertiary cancer centers, results may not be generally applicable to all types of clinical practice. Third, while this study is the largest regarding IMDC and SITs, the sample size in the combined group was relatively small, which limited the power in the subgroup analysis. Fourth, the limited data availability in retrospective chart review prohibited the accurate collection of cumulative dose and duration of steroid use through all IMDC episodes, and additional cancer therapy after ICI termination, which could potentially confound the results of cancer outcome and survival. Fifth, given the exclusion of patients treated with steroids alone, the specific impact of SIT on cancer outcome and OS could not be assessed. Sixth, we note a significant difference in cancer type, and ICI regimens among the two treatment groups and acknowledge that tumor biology, and different mechanism of action from specific ICI agents may play a role in both irAEs as well as cancer outcomes. Last, given the lack of standard guidelines for SITs use for IMDC, there were significant variations in the treatment regimens utilized by the treating physicians, which may have introduced biases to the study.

In conclusion, in a large retrospective two-center cohort, we observed that treatment with VDZ compared with IFX for IMDC led to comparable rates of colitis clinical remission, with shorter duration of steroid use, fewer hospitalizations and lower rate of IMDC recurrence, though with slower onset of action. Furthermore, higher doses of SIT were associated with better survival outcome, while more steroid exposure resulted in worse patient outcome. Future prospective randomized trials are needed to further confirm these observations and clarify the impact of different SITs on cancer outcome.

10.1136/jitc-2021-003277.supp6 Supplementary data

10.1136/jitc-2021-003277.supp7 Supplementary data

## Data Availability

Data are available on reasonable request.
