# Supplementary material for: Efficacy and safety of vedolizumab and infliximab treatment for immune-mediated diarrhea and colitis in patients with cancer: a two-center observational study
Source: J Immunother Cancer. 2021 Nov 17;9(11):e003277. doi: 10.1136/jitc-2021-003277 (PMC8601082; doi:10.1136/jitc-2021-003277)
Supplement: Supplementary data [file jitc-2021-003277supp001.pdf]

Supplemental Table 1. Patient selection in MDACC and MSKCC (*N*=184)

Center	Vedolizumab alone	Infliximab alone	Combined sequentially
MDACC	61	71	18
MSKCC	1	23	10

MDACC: The University of Texas MD Anderson Cancer Center
MSKCC: Memorial Sloan Kettering Cancer Center
At MSKCC, all patients treated with vedolizumab for biopsy-proven IMDC, who met inclusion criteria, were enrolled. A 2:1 matched cohort of biopsy-proven IMDC patients treated with infliximab was also included.

Supplemental Table 2. Patients' baseline demographic and clinical characteristics (*N*=184).

Characteristic	No. of patients (%)
Median age at IMDC– years (IQR), n=184	64 (51-73)
Male sex	118 (64)
White race	169 (92)
Median Charlson Comorbidity Index at IMDC, points. (IQR), n=184	8 (6-9)
Cancer type	
Melanoma	63 (34)
Genitourinary cancer (GU)	61 (33)
Lung cancer	31 (17)
Others*	29 (16)
Cancer Stage	
Stage III	33 (18)
Stage IV	151 (82)
Checkpoint inhibitor type	
CTLA-4	28 (15)
PD-(L)1	92 (50·00)
Combination	64 (35)
Cancer progression at IMDC	54 (29)
Median follow-up duration, mo. (IQR), n=184	14 (8-27)
Cancer progression at last follow up	84(46)
Non-GI organs involving adverse events†– no. (%)	
Skin	48 (26)
Endocrine	35 (19)
Pancreas	24 (13)
Liver	23 (12)
Musculoskeletal	18 (10)
Hematological	13 (7)
Lungs	11 (7)
Other‡	15 (8)
All-cause mortality	68 (37)

IQR denotes interquartile range, NSAID non-steroidal anti-inflammatory drug, PPI, proton pump inhibitor, GI gastrointestinal, GU genitourinary, CTLA-4 cytotoxic T lymphocyte antigen-4, and PD-(L) 1 programmed death-1/programmed death ligand 1. IMDC, immune-mediated diarrhea and colitis. *Other cancer types included GI/hepatobiliary cancer, head and neck/endocrine cancer, hematologic cancer, breast cancer, cervical cancer, sarcoma cancer. †GI adverse events were defined according-the

Common Terminology Criteria for Adverse Events version 5·0. ‡Other non-GI adverse events consisted of mucositis, fatigue, and eye toxicity.

Supplemental Table 3. IMDC characteristics of patients received different treatment regimen (*N*=184).

**Characteristic**	**Vedolizumab n=62**	**Infliximab n=94**	**Combined sequentially n=28**	***P***
Median time from ICI initiation to IMDC onset, days (IQR), n=184	104 (53-212)	96 (33-188)	108·5 (51-215)	0·068
Diarrhea grade, no. (%)				0·134
1-2	20 (32)	33 (35)	6 (21)	
3-4	42 (68)	61 (65)	22 (79)	
Colitis grade– no. (%)				0·086
1-2	30 (48)	57 (62)	15 (55)	
3-4	32 (52)	37 (39)	13 (46)	
Median duration of initial IMDC symptoms, days (IQR), n=184	56 (27-80)	50 (31-69)	63 (49-104)	0·056
Median duration of steroids for initial IMDC, days (IQR), n=184	35 (27-43)	50 (41-68)	44 (35-100)	<0·001
IV steroids, no. (%)	36 (58)	58 (62)	23 (83)	0·032
Number of steroid tapering attempts prior to SIT use, median (IQR), n=184	1 (1-3)	2 (2-3)	2 (1-3)	0·035
Number dose of SIT, no. (%)				<0·001
1-2	21 (34)	77 (82)	0 (0.00)	
≥3	41 (66)	17 (18)	28 (100·00)	
Doses of SIT, mean (SD)	3 (2)	2 (1)	6 (4)	<0·001
Median duration from IMDC onset to first dose of SIT, days (IQR), n=184	11 (9-48)	23 (19-37)	33 (22-62)	0·003
Hospitalization, no. (%)	40 (65)	67 (71)	21 (75)	0·284
Multiple hospitalizations, no. (%)	10 (16)	26 (28)	14 (50)	0·011
Median duration of hospitalization, days (IQR), n=104	11 (6-16)	14 (8-20)	16 (10-27)	0·013
Clinical remission, no. (%)	55 (89)	83 (88)	19 (68)	0·296
Recurrent IMDC, no. (%)	8 (14)	27 (29)	7 (25)	0·008
Steroid/IFX/VDZ associated Infection*, no. (%)	12 (19)	23 (25)	8 (29)	0·281

*Infections included: urinary tract infection, intestinal infection (E Coli), periodontitis, pneumonia, bacteremia, candida esophagitis.
IFX: infliximab; IMDC: immune mediated diarrhea and colitis; IQR: interquartile range; IV: intravenous; SIT: selective immunosuppressive therapy; VDZ: vedolizumab; SD: standard deviation;

Supplemental Table 4. Cancer progression among subgroups of patients treated with IFX and VDZ within the same time window (2017-2020). N=163

Zou F, *et al. J Immunother Cancer* 2021; 9:e003277. doi: 10.1136/jitc-2021-003277

**Characteristic**	**Vedolizumab n=62**	**Infliximab n=94**	**Combined sequentially n=28**	***P***
Cancer progression rate at IMDC onset, n=50	15 (24)	30(41)	5(18)	0·092
Cancer progression rate at last follow up, n=76	21 (34)	42(58)	12(43)	0·022
p	0·323	0·034	0·080	

IMDC: immune mediated diarrhea and colitis

Supplemental Table 5. Univariate and multivariate logistic regression analysis for risk factors of cancer progression.

	**Univariate analysis**			**Multivariate analysis**		
**Variable**	**Odds ratio**	**95% CI**	***P***	**Odds ratio**	**95% CI**	***P***
Charlson Comorbidity Index	1·20	1·04-1·39	0·012	1·21	1·03-1·42	0·020
Cancer type						
Genitourinary cancer	0·47	0·19-1·14	0·095	0·37	0·13-1.02	0·667
Melanoma	0·79	0·31-1·91	0·595	0·18	0·06-0·53	0·005
Lung cancer	0.76	0·28-2·10	0·599	0.43	0·13-1·41	0·875
Other		Reference				
Cancer stage IV vs. III	1·87	0·58-4·13	0·120			
Duration of ICI treatment	0·99	0·98-1·00	0·067			
ICIs doses	0·96	0·92-1·01	0·069			
ICIs type						
anti-CTLA-4		Reference				
anti-PD-(L)-1	1·58	0·66-3·79	0·306			
Combination	1·69	0·68-4·22	0·261			
Duration from ICI to colitis onset	0·99	0·97-1·00	0·078			
Duration of colitis symptoms	0·99	0·98-1·01	0·102			
Diarrhea grade 3-4 vs. 1-2	1·37	0·77-2·46	0·289			
Colitis grade 3-4 vs. 1-2	1·66	0·88-3·12	0·119			
Overall duration of steroids	1·01	1·00-1·02	0·007			
IV steroids	1.41	0·77-2·59	0·271			
Number of steroid tapering attempts prior to SIT use	1·17	0·86-1·60	0·322			
Type of SIT						
Vedolizumab		Reference			Reference	
Infliximab	2·32	1·19-4·49	0·013	5·24	2·33-11·77	<0·001
Combined sequentially	1·46	0·59-3·65	0·414	2·07	0·76-5·58	0·817
No. of SIT doses	0·80	0·68-0·94	0·070			
Duration of IMDC to last follow-up	0·89	0·86-0·93	0·332			

CTLA-4: cytotoxic T-lymphocyte-associated protein 4; ICI: immune checkpoint inhibitor, IMDC: immune mediated diarrhea and colitis; IV: intravenous; PD-(L)-1: programmed cell death 1 protein/ligand; SIT, selective immunosuppressive therapy

Supplemental Table 6. Multivariate Cox regression analysis for overall survival.

Characteristic	Hazard Ratio	95% CI	*P*
Charlson Comorbidity Index	1·18	1·04-1·34	0·009
ICI type			
PD-(L)1		Reference	
CTLA-4	0·28	0·12-0·67	0·004
Combination	0·92	0·54-1·56	0·745
SIT type			
Vedolizumab	Reference		
Infliximab	2·04	1·15-3·62	0·014
Combined Sequentially	1·17	0·48-2·82	0·734

CTLA-4: cytotoxic T-lymphocyte-associated protein 4; ICI: immune checkpoint inhibitor; IMDC: immune mediated diarrhea and colitis; PD-(L)-1: programmed cell death 1 protein/ligand; SIT: selective immunosuppressive therapy.

Zou F, *et al. J Immunother Cancer* 2021; 9:e003277. doi: 10.1136/jitc-2021-003277
