# Supplementary material for: Efficacy and safety of vedolizumab and infliximab treatment for immune-mediated diarrhea and colitis in patients with cancer: a two-center observational study
Source: J Immunother Cancer. 2021 Nov 17;9(11):e003277. doi: 10.1136/jitc-2021-003277 (PMC8601082; doi:10.1136/jitc-2021-003277)
Supplement: Supplementary data [file jitc-2021-003277supp002.pdf]

Figure legends:

Supplemental Figure 1. Cancer outcome stratified by SIT used at different time points.

Supplemental Figure 2: Overall survival curves between (A) vedolizumab (VDZ) alone, infliximab (IFX) alone, or combined selective immunosuppressive therapies sequentially (SIT); (B) ≤2 and >2 steroid tapering attempts groups; (C) 1-2 doses of selective immunosuppressive therapy (SIT) and ≥3 doses SIT groups. IMDC, immune-mediated diarrhea and colitis. Patients who had follow-up duration longer than 45 months were administratively censored at 45 months.

Supplemental Figure 3. Subgroup analysis of overall survival (OS) curves from onset of immune-mediated diarrhea and colitis in patients within the same follow-up time window (2017-2020) (A) among different selective immunosuppressive therapy (SIT) regimens; between vedolizumab (VDZ) and infliximab (IFX) treated patients with; (B) genitourinary cancer; (C) lung cancer; and (D) melanoma. Patients who had follow-up duration longer than 45 months were administratively censored at 45 months.

Supplemental Figure 4. Overall survival curves from onset of immune-mediated diarrhea and colitis between vedolizumab (VDZ) and infliximab (IFX)-treated patients with (A) genitourinary cancer; (B) lung cancer; and (C) melanoma. Patients who had follow-up duration longer than 45 months were administratively censored at 45 months.

Supplemental Figure 5. Infection associated with immunosuppressant use. (A) Comparison between short and long steroid treatment duration in different treatment groups; (B) comparison between fewer and more steroid treatment courses in different treatment groups.
