# Supplementary figures and images for: Efficacy and safety of vedolizumab and infliximab treatment for immune-mediated diarrhea and colitis in patients with cancer: a two-center observational study
Source: J Immunother Cancer. 2021 Nov 17;9(11):e003277. doi: 10.1136/jitc-2021-003277 (PMC8601082; doi:10.1136/jitc-2021-003277)

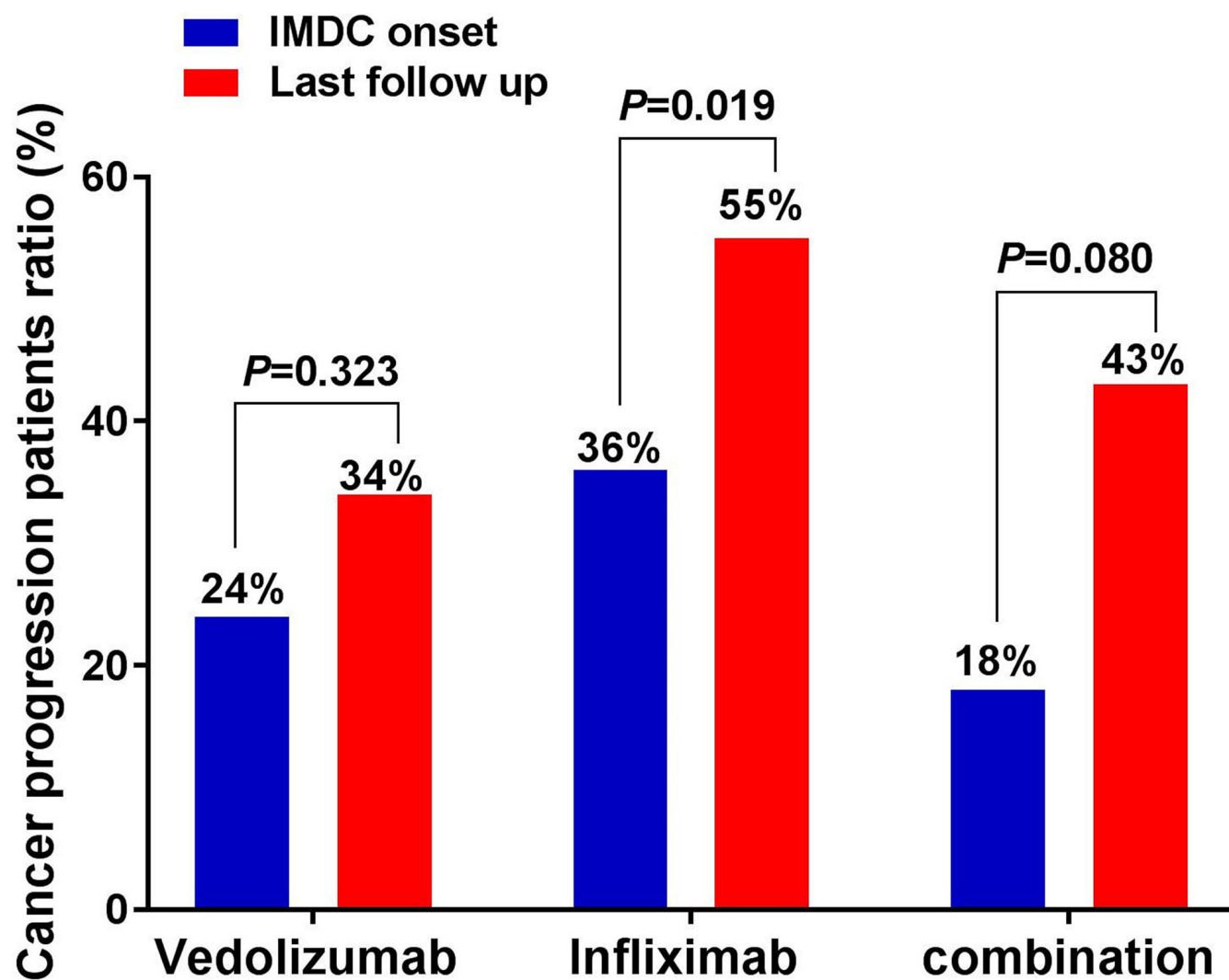

Supplement: Supplementary data [file jitc-2021-003277supp003.pdf]

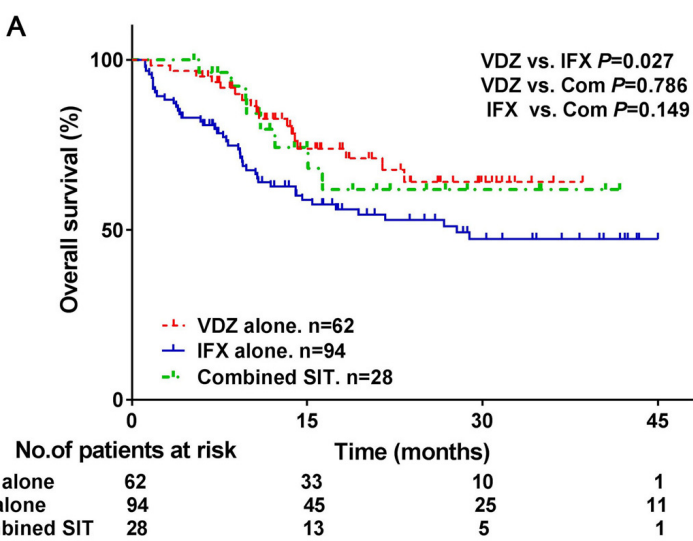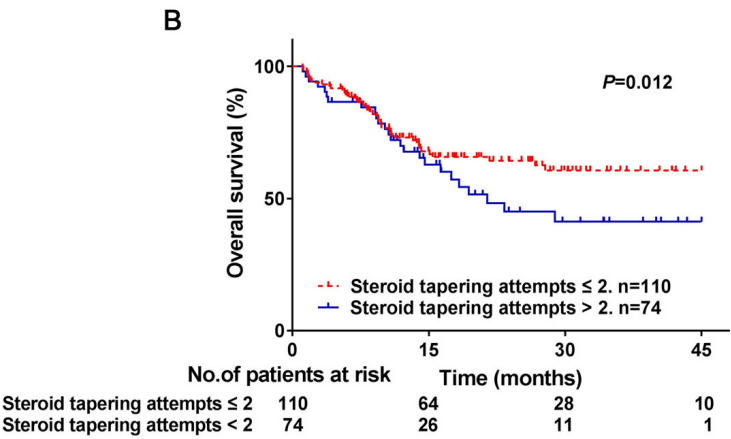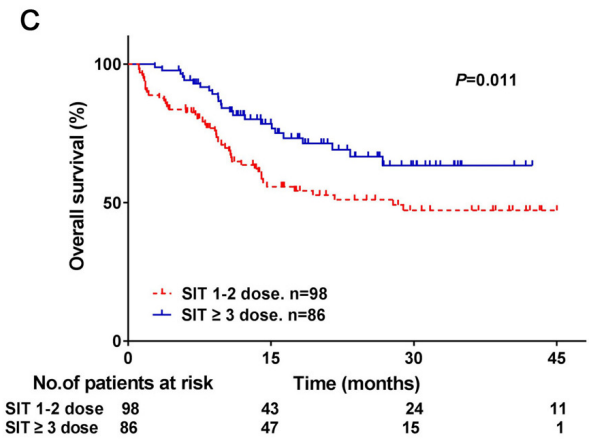

Supplement: Supplementary data [file jitc-2021-003277supp004.pdf]

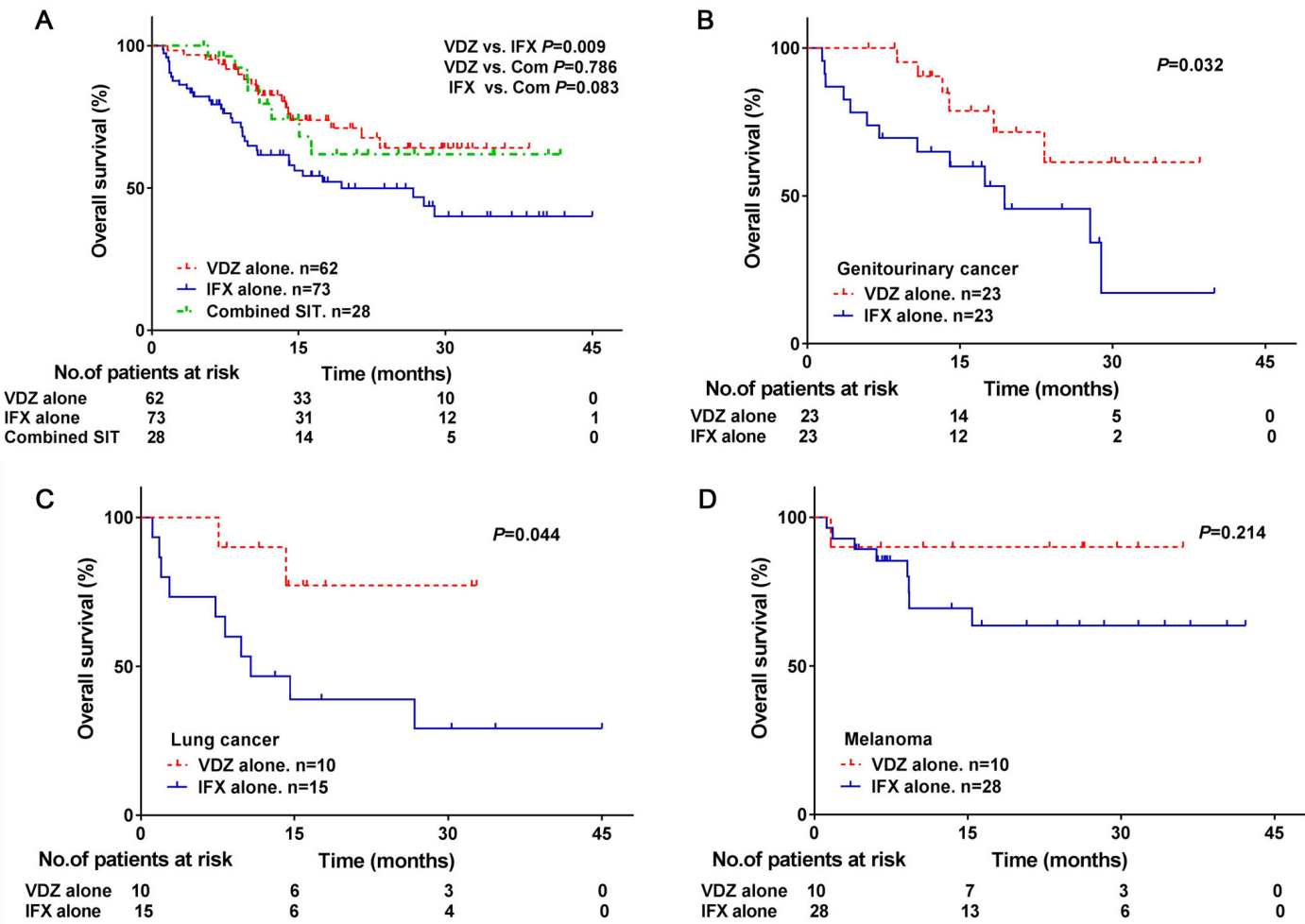

Supplement: Supplementary data [file jitc-2021-003277supp005.pdf]

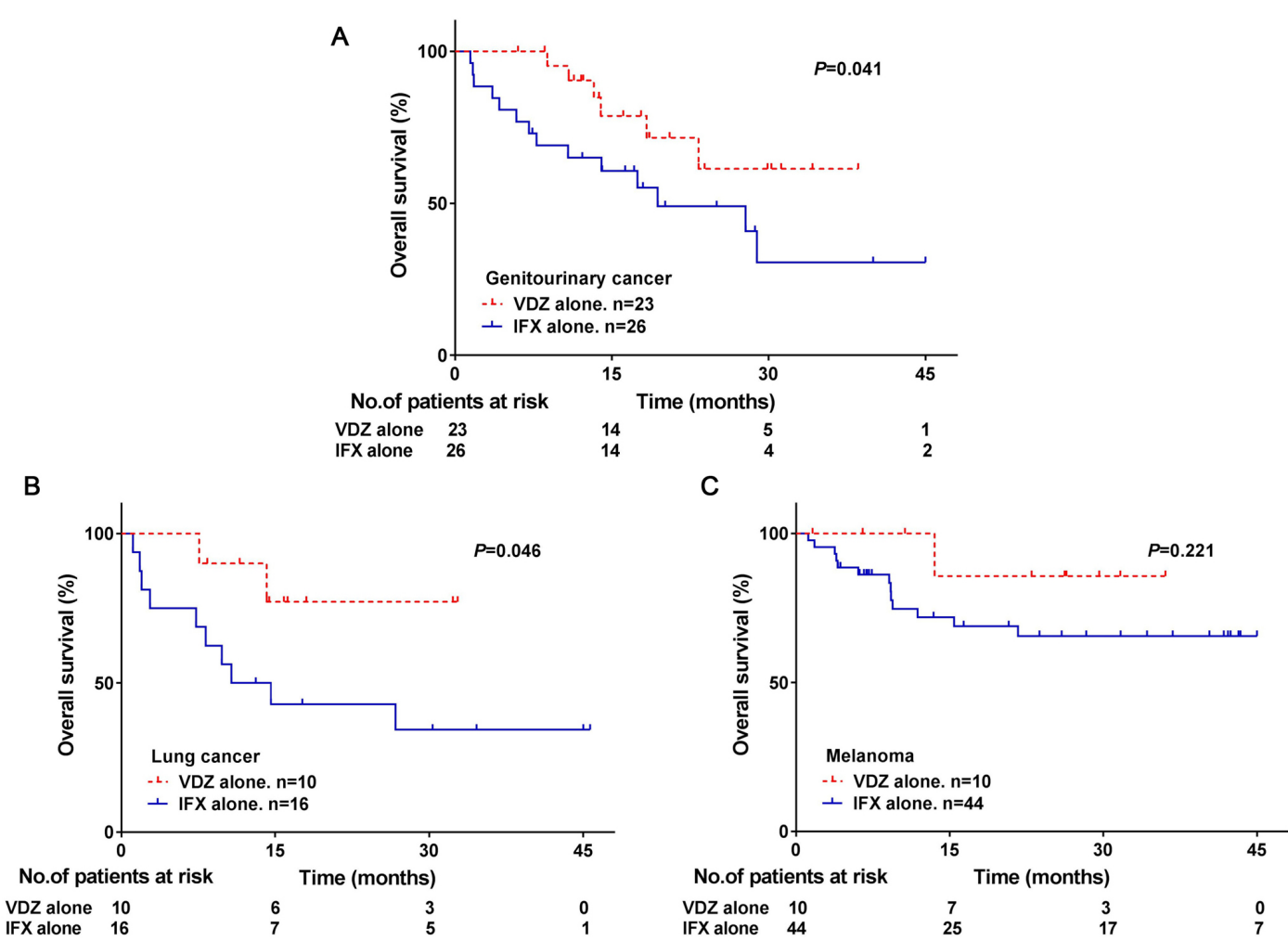

Supplement: Supplementary data [file jitc-2021-003277supp006.pdf]

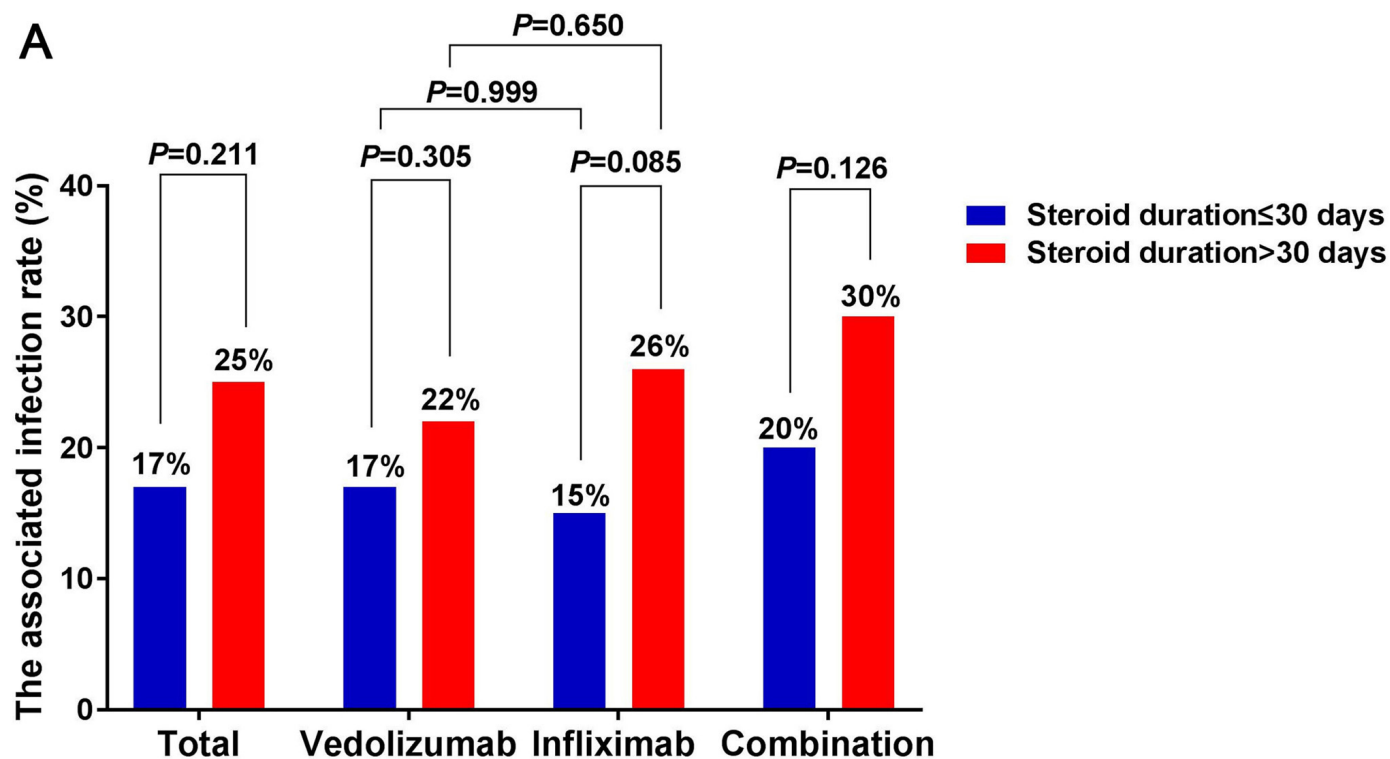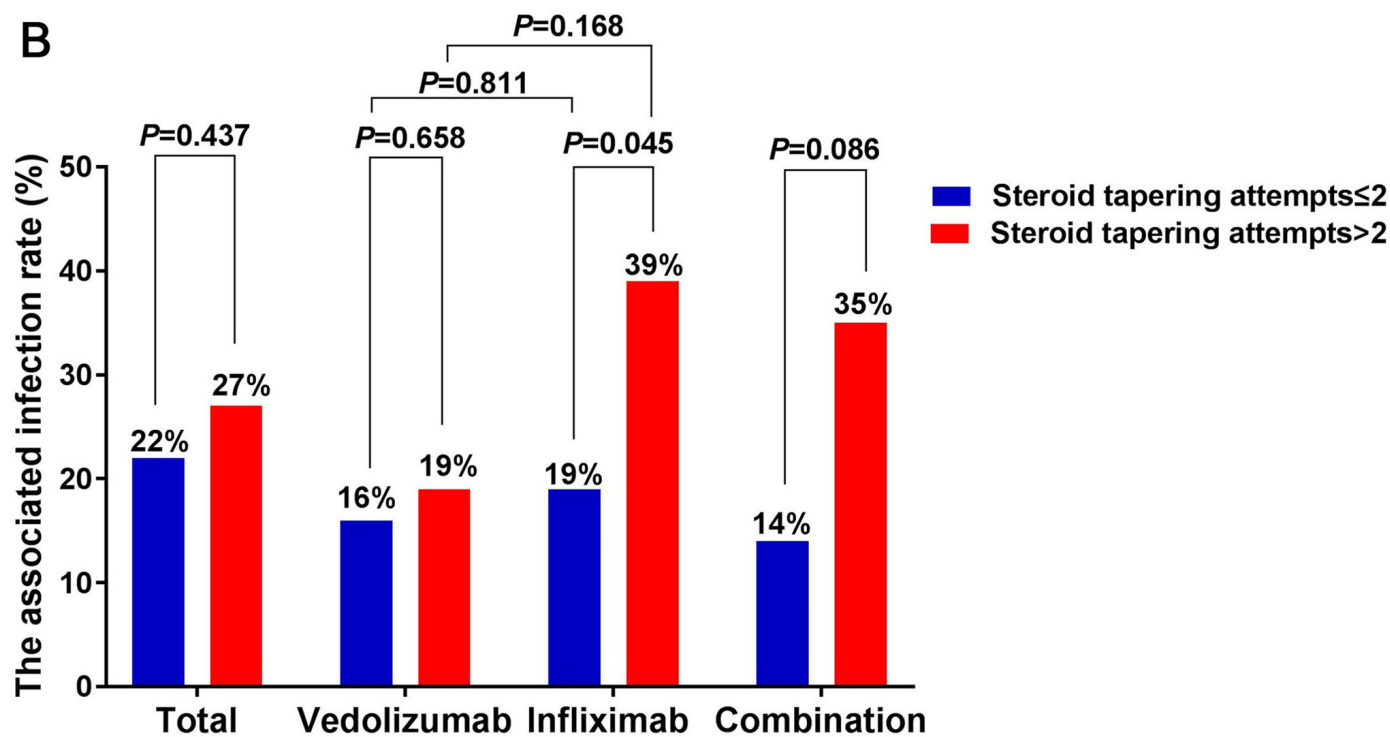

Supplement: Supplementary data [file jitc-2021-003277supp007.pdf]
